# Dual‐branch Transformer for semi‐supervised medical image segmentation

**DOI:** 10.1002/acm2.14483

**Published:** 2024-08-12

**Authors:** Xiaojie Huang, Yating Zhu, Minghan Shao, Ming Xia, Xiaoting Shen, Pingli Wang, Xiaoyan Wang

**Affiliations:** ^1^ The Second Affiliated Hospital School of Medicine Zhejiang University Hangzhou China; ^2^ Zhejiang University of Technology Hangzhou China; ^3^ Stomatology Hospital School of Stomatology Zhejiang University School of Medicine Hangzhou China

**Keywords:** dual‐branch structure, medical image segmentation, semi‐supervised learning, Transformer

## Abstract

**Purpose:**

In recent years, the use of deep learning for medical image segmentation has become a popular trend, but its development also faces some challenges. Firstly, due to the specialized nature of medical data, precise annotation is time‐consuming and labor‐intensive. Training neural networks effectively with limited labeled data is a significant challenge in medical image analysis. Secondly, convolutional neural networks commonly used for medical image segmentation research often focus on local features in images. However, the recognition of complex anatomical structures or irregular lesions often requires the assistance of both local and global information, which has led to a bottleneck in its development. Addressing these two issues, in this paper, we propose a novel network architecture.

**Methods:**

We integrate a shift window mechanism to learn more comprehensive semantic information and employ a semi‐supervised learning strategy by incorporating a flexible amount of unlabeled data. Specifically, a typical U‐shaped encoder‐decoder structure is applied to obtain rich feature maps. Each encoder is designed as a dual‐branch structure, containing Swin modules equipped with windows of different size to capture features of multiple scales. To effectively utilize unlabeled data, a level set function is introduced to establish consistency between the function regression and pixel classification.

**Results:**

We conducted experiments on the COVID‐19 CT dataset and DRIVE dataset and compared our approach with various semi‐supervised and fully supervised learning models. On the COVID‐19 CT dataset, we achieved a segmentation accuracy of up to 74.56%. Our segmentation accuracy on the DRIVE dataset was 79.79%.

**Conclusions:**

The results demonstrate the outstanding performance of our method on several commonly used evaluation metrics. The high segmentation accuracy of our model demonstrates that utilizing Swin modules with different window sizes can enhance the feature extraction capability of the model, and the level set function can enable semi‐supervised models to more effectively utilize unlabeled data. This provides meaningful insights for the application of deep learning in medical image segmentation. Our code will be released once the manuscript is accepted for publication.

## 1 INTRODUCTION

In the medical field, it is often necessary for doctors to analyze various medical images to fulfill diagnostic and treatment needs. However, medical images often suffer from issues such as blurriness and low signal‐to‐noise ratio due to the limitations of medical imaging devices, which may result inaccurate diagnosis.^1^ The primary goal of medical image segmentation is to delineate regions of interest in the images, such as specific organ regions or tumors, which can greatly facilitate diagnosis.^2^ Recently, deep learning has shown increasingly significant performance in many image segmentation tasks. Although deep neural network models have shown excellent performance in image segmentation, their current success relies heavily on large annotated datasets. Unlike natural images, annotating medical images is not only time‐consuming and labor‐intensive but also requires expert knowledge.^3^ Additionally, privacy concerns regarding patient data must be taken into account. These factors make it challenging to obtain medical image datasets especially that with fine annotations.^4^ Therefore, how to train a precise and highly generalizable neural network with limited data is a problem worth studying.

Convolutional neural network (CNN) is popular for its powerful feature representation capability and have made remarkable achievements in computer vision (CV).^5^, ^6^, ^7^, ^8^ However, due to the limitation of receptive fields, convolutional neural networks are more likely to focus on local details and ignore contextual information in the process of downsampling, which makes their performance unsatisfactory. Unlike CNNs, Transformer's self‐attention mechanism can make good use of global information,^9^, ^10^, ^11^ so researchers have put a lot of effort into exploring its adaptability in CV. Among them, Swin Transformer^12^ builds a hierarchical Transformer by introducing the hierarchical construction method commonly used in CNNs. In Swin Transformer, self‐attention computation is performed based on windows, which enhances the model's local feature representation and reduces computational costs. At the same time, by shifting the windows, the advantage of Transformers in learning long‐range dependencies is also preserved. Currently, Swin Transformer performs exceptionally well in a variety of CV tasks.

Semi‐supervised learning approaches are presented to address the issue of limited labeled data, with the basic idea of training neural networks using a limited amount of labeled data and an arbitrary amount of unlabeled data.^13^ Implementing this idea is challenging because unlabeled data, lacking precise annotations from professionals, may be affected by noise, artifacts or other factors, leading to differences in image quality and content. These factors can interfere with model learning, resulting in misleading features.^14^ Labeled data, on the other hand, provides accurate semantic information, enabling the model to learn the true features and structures better. In semi‐supervised learning, to effectively utilize unlabeled data during model training, a simple and intuitive approach is to assign pseudo‐labels to the unlabeled data and then train the model using labeled and pseudo‐labeled data together.^15^, ^16^ Pseudo‐labels are typically generated iteratively, where the model's predictions on unlabeled data serve as its pseudo‐annotations, and the quality of pseudo‐labels is improved through repeated iterations. However, despite showing certain performance in semi‐supervised learning with pseudo‐labeling,^17^, ^18^, ^19^, ^20^, ^21^, ^22^ the annotations generated by the model can still be noisy, which can have a undesirable effect on the subsequent model training.^23^ To mitigate the noise impact of pseudo‐labels, Luo et al.^32^ first proposed utilizing the level set function to establish a dual‐task consistent semi‐supervised framework. This framework jointly predicts pixel‐level segmentation maps and geometric‐aware level set representations of segmentation targets. By deriving segmentation maps from the level set function and establishing task‐level consistency loss between the derived segmentation maps and directly predicted segmentation maps, high‐quality pseudo‐labels can be obtained. Additionally, Chen et al.^41^ constructed a consistency joint learning framework using the level set function to encourage consistency between segmentation results and predicted signed distance maps generated by the level set function. However, these two works are designed only for 3D medical image segmentation, but given the outstanding performance of the level set function in semi‐supervised learning, we incorporate this consistency loss into our model.

By fully integrating the respective strengths of Swin Transformer and level set functions in deep learning, a Transformer‐based dual‐branch semi‐supervised image segmentation method is proposed in this paper. In this method, the dual‐branch Swin modules serve as the backbone for feature extraction. By setting different window sizes for each branch, this model can integrate features of different scales in the image, enabling it to learn richer image features. Additionally, considering that the level set function can effectively describe the shape of object boundaries in images, providing relatively stable boundaries even in the presence of noise or poor image quality, we use these boundaries as geometric constraints to establish consistency between global function regression and pixel classification tasks. This approach enhances the quality of pseudo‐labels in semi‐supervised learning, thereby improving the quality of medical image segmentation.

**FIGURE 1:**
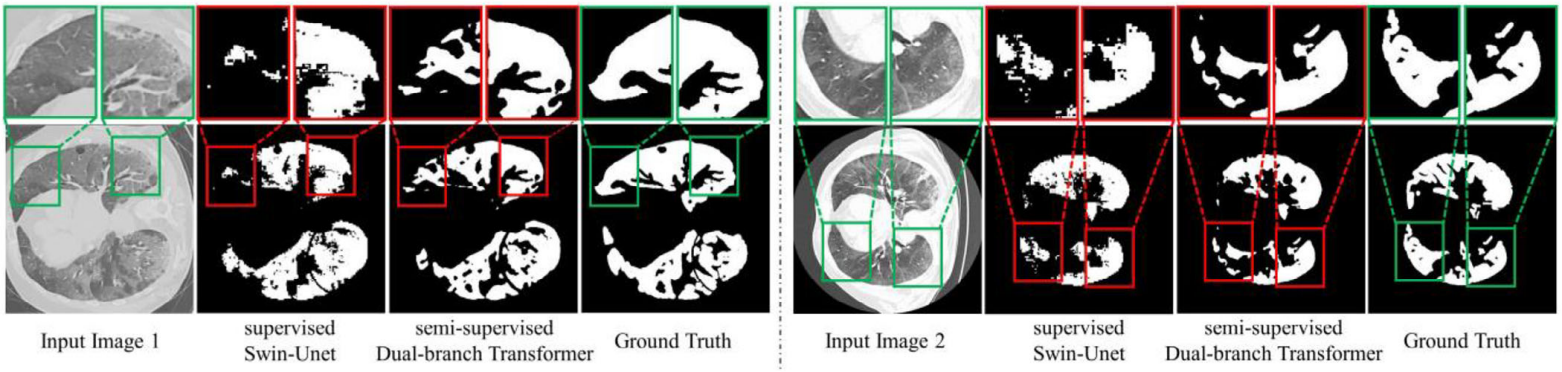
Examples of the segmentation performance of the proposed semi‐supervised Dual‐branch Transformer and the fully supervised model Swin‐Unet on the COVID‐19 CT dataset. For semi‐supervised learning, 20% of the training set are labeled, and the remaining 80% are unlabeled. The first row shows an enlarged view of the boxed region in the second row. It can be seen that our semi‐supervised method provides a more precise characterization of shape features due to the incorporation of geometric constraints.

We selected two instances from the COVID‐19 CT dataset and visualized the segmentation results of these two instances using our proposed semi‐supervised method and the fully supervised model Swin‐Unet,^24^ respectively. During training, we used 80% of the unlabeled data for our model, while Swin‐Unet used fully labeled data. The visual results indicate that our approach can still achieve good segmentation results even in the absence of a large amount of labeled data compared to the fully supervised transformer‐based model. As seen on the left in Figure 1, our method demonstrates superior capability in capturing details. Figure 1 on the right demonstrates that our method provides a more precise characterization of shape features due to the incorporation of geometric constraints. Specifically, our contributions are as follows:

(1) We propose a semi‐supervised learning method that adopts two different training strategies based on the availability of labels. It establishes consistency between function regression tasks and pixel classification tasks, allowing the incorporation of geometric constraints into the model training process. This approach leverages all available data effectively to improve the model's generalization ability.
(2) We introduce a dual‐branch structure in the feature extraction stage. One branch consists of Swin modules with small windows, which are more focused on capturing smaller scale features. The other branch consists of Swin modules with large windows, which increase the model's receptive field and are more focused on capturing smaller scale features. The dual‐branch structure achieves precise segmentation by integrating features from different perspectives.
(3) We conduct extensive experiments on two datasets with distinct characteristics and compare our method with several fully supervised and semi‐supervised models. The results show that our method outperforms not only the semi‐supervised models but also the fully supervised models.

## 2 RELATED WORK

### 2.1 Transformers in computer vision

Due to the prominent accomplishments of Transformers in natural language processing (NLP), research in CV has also begun to focus on models based on transformers. ViT,^25^ as the first Transformer model used in CV, benefits from the design of image patch and positional encoding. It successfully transplants Transformers from NLP to CV and has achieved excellent outcomes in various tasks. Since then, ViT has carved out a niche in CV due to its powerful ability to learn global features. Subsequently, more and more models have followed in the footsteps of ViT, making Transformers shine in the field of image analysis.^26^, ^27^, ^28^ For example, MAE^29^ is a pre‐training method that models the long‐range dependencies of input images using Transformers through masking. It has achieved notable achievements in various downstream tasks and has become a new paradigm for pre‐training models in CV. Swin Transformer,^12^ with its hierarchical structure and unique shift window design, enables Transformers to be more flexible in dealing with i‐age data of various scales and improves computational efficiency.

In medical image processing, MedT^30^ proposes a gated axial attention model and a local‐global training strategy. Swin‐Unet^24^ uses Swin Transformer blocks as the basic units for feature representation and long‐range semantic information interaction learning, while DS‐TransUNet^31^ integrates hierarchical Swin Transformers into the encoder‐decoder structure of U‐Net. Although these models have obtained a commendable performance, there is still great potential for improvement in medical image processing, as medical images often contain many fine‐grained features that a single Transformer‐based model may not fully capture.

### 2.2 Semi‐supervised learning for medical image segmentation

Semi‐supervised learning aims to train neural network models with higher effectiveness by utilizing both labeled and unlabeled data. Due to the difficulty of collecting labeled medical image data, semi‐supervised learning for medical image segmentation has gained significant attention. Several semi‐supervised learning models have been designed and have made striking progress in medical image segmentation.^32^, ^33^, ^34^, ^35^

One widely used approach is to use pseudo‐labeling generated by the model to assign annotations to unlabeled data. This method combines pseudo‐labeled data with labeled data to train the model and iteratively improves the quality of the pseudo‐labeling. Some studies have demonstrated the effectiveness of this approach. However, since only a very small quantity of labeled data is employed at the beginning of model training, the outputs are prone to noise, leading to noisy pseudo‐labels and ultimately affecting the model's performance.

In recent years, semi‐supervised learning with regularization terms has become popular.^36^, ^37^, ^38^, ^39^ It includes unlabeled data into the training process by generating unsupervised regularization signals during training. This approach effectively leverages unlabeled data while avoiding the noise issue caused by generating pseudo‐labels. There are various methods to implement unsupervised regularization. For example, Li et al.^40^ developed a multi‐task network with adversarial regularization for shape‐aware constraints. Luo et al.^32^ combined level set function regression tasks with segmentation tasks to achieve consistency in semi‐supervised learning. Chen et al.^41^ also used a framework of joint learning with consistency to encourage consistency between segmentation results and signed distance map predictions.

Inspired by the aforementioned research, method proposed in this paper establishes consistency between a global function regression task and a pixel classification task at the output stage of the model. Leveraging the advantages of Transformers in global feature learning and utilizing the window‐based advantages of the Swin module, a dual‐branch structured model is designed. This model can simultaneously focus on both local and global features of the image. By adding set constraints to the loss function, the segmentation capability of the semi‐supervised model is improved.

## 3 METHOD

### 3.1 Overall architecture

**FIGURE 2:**
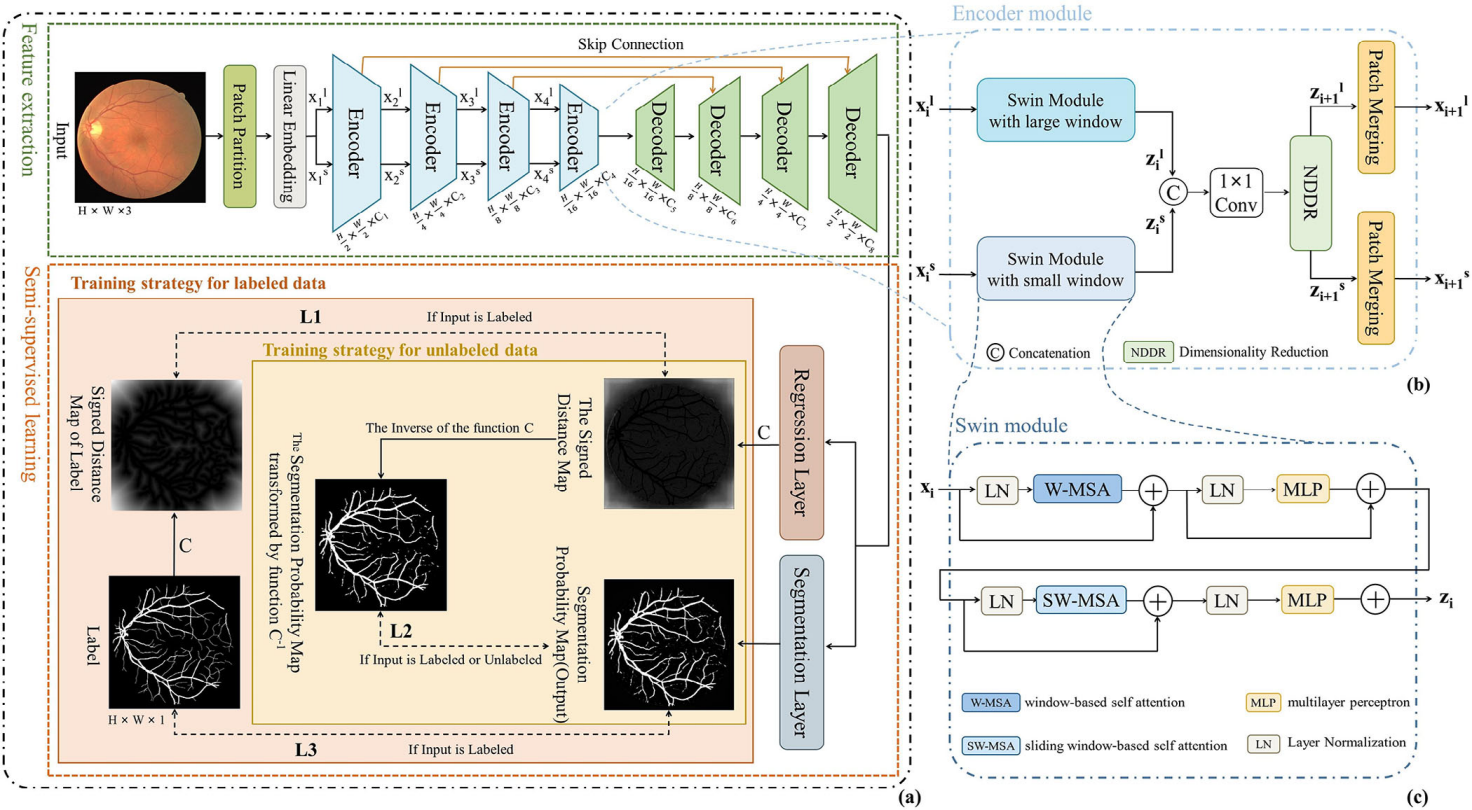
Overall Architecture. (a) shows the training strategy and loss functions for semi‐supervised learning. L1 refers to the distance loss function that is based on the signed distance maps of the labels and the signed distance maps of the segmentation maps. L2 is a dual‐task consistency loss that is defined between the inverse transformation of signed distance maps and the segmentation probability maps. L3 is a loss function that is based on the labels and the segmentation probability maps. C and C^−1^ represent the level set function and its inverse function, respectively. (b) Illustrates the specific dual‐branch structure within each encoder. And (c) shows the structure of the Swin Module.

The overall structure of this method is shown in Figure 2, and it can be divided into two main parts: feature extraction and semi‐supervised learning. In the feature extraction part, a traditional encoder‐decoder structure is adopted. Then, the training effectiveness of semi‐supervised learning is enhanced by introducing a regression layer and a segmentation layer after the feature extraction part.

The feature extraction part consists of four encoders and corresponding decoders, each corresponding to one of the four encoders. Each encoder fully leverages the advantages of Transformers in global feature extraction and combines features with different receptive field sizes in a dual‐branch form. During the encoding process, the size of input gradually decreases in spatial dimensions as it passes through each encoder. In the decoder part, features extracted from final encoder are upsampled through deconvolution to increase their spatial scale step by step, ensuring that the output of the feature extraction part has the same size as the input image. Skip connections are added between encoders and decoders to combine the features extracted by each encoder with the upsampled features obtained by the corresponding decoder.

The output obtained from the decoder is then sent to the output stage, which consists of two branches too. The first branch consists of regression layer, which generates a signed distance map. The second branch consists of segmentation output layer, responsible for producing the segmentation result map. The outputs of both branches interact with each other, establishing consistency between the global function regression task and the pixel classification task. This allows the dual‐branch Transformer backbone to be used in semi‐supervised image segmentation tasks to obtain richer features and more accurate segmentation results.

### 3.2 Feature extraction

Feature extraction part follows a classical encoder‐decoder structure. In the encoder stage, each encoder consists of two parallel Swin modules. The Swin module retains the basic structure of the Transformer encoder but replaces the multi‐head self‐attention calculation with window‐based multi‐head self‐attention calculation. It divides the image into non‐overlapping windows of size n×n (where *n* represents the number of patches) and performs self‐attention calculation within each window to obtain the feature information within the window. Then, sliding window attention calculation is performed to allow information interaction between windows. The formula for the Swin module is as follows:

(1) $$Z_{i}=\mathrm{WMSA}\left(\mathrm{LN}\left(Z_{i-1}\right)\right)+Z_{i-1},$$

(2) $$Z_{i}=\mathrm{MLP}\left(\mathrm{LN}\left(Z_{i}\right)\right)+Z_{i},$$

(3) $$Z_{i+1}=S\mathrm{WMSA}\left(\mathrm{LN}\left(Z_{i}\right)\right)+Z_{i},$$

(4) $$Z_{i+1}=\mathrm{MLP}\left(\mathrm{LN}\left(Z_{i+1}\right)\right)+Z_{i+1},$$

where, WMSA(·) represents window‐based self attention, LN(·) represents the linear layer, SWMSA(·) represents the sliding window‐based self attention, MLP(·) represents multilayer perceptron and $Z_{i-1}$ to $Z_{i+1}$ represent the consecutive outputs within the Transformer. The two parallel Swin modules have different window sizes, which means they capture different information through window attention. The small window has smaller receptive field, which is more focused on detailed information, while the large window has larger receptive field, which can capture larger scale features. To combine the different information from the different branches, a 1×1 convolutional layer is used to fuse the branch outputs before sending them back to their respective branches. The fused feature maps then undergo a slice fusion operation before entering the next encoder stage. Patch merging splits the input feature maps in the spatial dimension and fuses them in the channel dimension. This operation reduces the size of the feature maps in length and width, gradually decreasing the scale of the feature maps during the encoding process and allowing the Transformer encoder to capture multi‐scale information.

The overall workflow in the encoder stage is as follows: Before the first encoder, the input image is first divided into one‐dimensional features using a patch partition layer and a linear embedding layer. These features are then separately input into two Swin modules with different window sizes for feature extraction. The outputs of the two branches are fused and exchanged through a 1×1 convolution, and then they are individually input into the next encoder stage. In the second to fourth encoders, the traditional pooling layers are replaced by patch merging operations to reduce the dimensionality of features. The reduced features are then passed on to next encoder.

In the decoder stage, each decoder first upsamples the input features through deconvolution to obtain outputs of the same size as the corresponding encoder stage. Considering that the encoding process compresses and loses a large amount of detailed information, skip connections are introduced to concatenate the decoder outputs with the feature maps obtained from the corresponding encoder. This allows better fusion of shallow positional information with deep semantic information, facilitating the generation of more precise segmentation masks. The concatenated features then undergo two 1×1 convolutions to fuse information in both channel and spatial dimensions, aiming to obtain higher‐resolution features. After passing through four decoders, we obtain a feature map with the same length and width as the input, and the channel size is 1.

### 3.3 Semi‐supervised learning and loss function

In the Semi‐supervised learning part, two branches are designed to transform the feature maps obtained from the feature extraction part into different types of outputs. One branch is the segmentation branch, which directly feeds the feature maps into an activation function to generate a segmentation probability map. The other branch contains of a regression layer processing the feature maps through a linear layer and a level set function C to generate a signed distance map. The level set function C is capable of capturing geometric contours and distance information, and its expression is as follows:

(5) $$C=\left\{\begin{array}{c} -\underset{y\in \partial T}{\inf}\left||x-y\right|{|}_{2},x\in T_{\mathrm{in}}\\ 0,x\in \partial T\\ +\underset{y\in \partial T}{\inf}\left||x-y\right|{|}_{2},x\in T_{\mathrm{out}} \end{array}\right.$$

where x and y represent two different pixels in the segmentation map, ∂T represents the precise contour between the interior and exterior in segmentation target, and $T_{\mathrm{in}}$ and $T_{\mathrm{out}}$ represent the interior and exterior of the target contour, respectively. The smoothing approximation to the inverse of the function C, denoted as $C^{-1}$, is employed to convert a signed distance map into a segmentation probability map. $C^{-1}$ is expressed as follows:

(6) $$C^{-1}=\frac{1}{1+e^{-kz}},z\in C,$$

where z is the signed distance value at pixel x and k is a factor as large as possible.

Specifically, different training strategies are adopted depending on whether the current input has label. When the input is labeled data, label of the input will transform into a signed distance map using the level set function C, and the regression layer is trained based on this transformed map. At the same time, a loss function is computed between the original label and the output of the segmentation layer, and the output of the regression layer is transformed back into the corresponding segmentation probability map using the inverse function $C^{-1}$, which is then used to compute a loss function with the output of the segmentation layer. In this case, the total loss function $L_{\mathrm{labeled}}$ consists of three parts:

First of all, the loss function between the signed distance map of the regression layer and the signed distance map obtained by converting the label through the function C defined as L1:

(7) $$L1(x,y)=\sum_{x_{i},y_{i}\in D}\left|\right|f_{1}\left(x_{i}\right)-C\left(y_{i}\right)|{|}^{2},$$

where *x*, *y* represent the outputs of the labeling and regression layers respectively, $f_{1}\left(x_{i}\right)$ is the signed distance map of the regression layer output, and $C(y_{i})$ is the signed distance map obtained by transforming the labeling by the function C.

Secondly, a dual‐task consistency loss L2 is also defined for the segmentation probability map obtained by implementing $C^{-1}$ to the output of the regression layer and the segmented probability map generated from the other branch to enhance the consistency between the transformed map of the regression layer and the segmented output layer. L2 is expressed as:

(8) $$L2(x)=\sum_{x_{i}\in D}\left|\right|f_{2}\left(x_{i}\right)-C^{-1}\left(x_{i}\right)|{|}^{2},$$

where *x* is the output from the backbone network, $f_{2}\left(x_{i}\right)$ represents the segmentation probability prediction of the segmentation layer, and the transformed map of the regression layer is represented by $C^{-1}\left(x_{i}\right)$.

Finally, the common cross‐entropy loss function L3 is used as the loss function for the segmentation probability maps of the label and the output of the segmentation layer, L3 is expressed as follows:

(9) $$L3(f)=-\frac{1}{p}\sum_{i=1}^{p}\log f_{i}\left(y_{i}*\right),$$

where p is the number of pixels in one image, $y_{i}^{*}$ is the category of pixel in the label, $f_{i}\left(y_{i}^{*}\right)$ is the probability estimate of the segmentation probability map's pixel *i*, and f is a vector of all outputs of $f_{i}\left(y_{i}\right)$.

To summarize, the total loss function for labeled data can be expressed as:

(10) $$L_{\mathrm{labeled}}=L1+L2+L3.$$

When the input is unlabeled data, its loss function is only the loss between the two output branches. $L_{\mathrm{unlabeled}}$ can be expressed as follows:

(11) $$L_{\mathrm{unlabeled}}=L2(x)=\sum_{x_{i}\in D}\left|\right|f_{2}\left(x_{i}\right)-C^{-1}\left(x_{i}\right)|{|}^{2},$$

## 4 EXPERIMENTS

### 4.1 Datasets and pre‐processing

The proposed semi‐supervised method and network architecture were evaluated on two datasets: the COVID‐19 CT dataset and the DRIVE dataset.

The COVID‐19 CT dataset^52^ consists of 100 axial CT images from over 40 patients, collected by the Italian Medical Society. In this dataset, the images are segmented into three labels: ground‐glass opacity, consolidation, and pleural effusion. Two abnormal images with completely black content were removed from the dataset. Experiments on this dataset utilizes 50 images for training and 48 images for testing. The preprocessing followed the same procedures as Inf‐net^42^ and SSA‐Net,^19^ which include adjusting the grayscale values of the lung window and cropping the images to a size of 512 × 512.

The DRIVE dataset^53^, ^54^ is a publicly available medical image dataset used for studying automatic segmentation algorithms for retinal images. It contains 40 high‐resolution digital retinal images, and 20 of them were selected for training, and the remaining 20 were used for testing in this study. Each image has a resolution of 768 × 584 pixels, with a pixel size of 0.0048 × 0.0048 mm. The segmentation masks, which identify the blood vessels in the retinal images, were manually annotated by professionals. The preprocessing step for this dataset involved resizing the images to 512 × 512.

#### 4.1.1 Implementation details

Our work is implemented with PyTorch. The small window size in our dual‐branch Swin module is set to 7, while the large window size is set to 14. The total parameter count of the model is 94.07 M. The network is trained for 8000 iterations on an NVIDIA 2080TI GPU, using Stochastic Gradient Descent (SGD) as the optimizer with an initial learning rate of 1e−2 and weight decay of 1e−4. Generally, training one epoch on the COVID‐19 CT dataset takes less than 1 min, and the network can be trained to convergence in less than 1 h. For the semi‐supervised image segmentation experiments, the labels were set according to the standard setting of conventional methods. In the training set, 20% the data were labeled, and the remaining 80% were unlabeled. Additionally, when training the DRIVE dataset, another retinal dataset called IDRiD^43^ was introduced. Forty 512 × 512 sized retinal images from the IDRiD dataset were included as unlabeled data in the training set of the DRIVE dataset. Finally, standard data augmentation techniques were applied to all datasets, including rotation, translation, scaling, flipping, and random cropping. In the comparison of methods, fully supervised methods were trained using the entire annotated training dataset. For the COVID‐19 CT dataset, a total of 50 labeled data samples were used to train these fully supervised models. For the DRIVE dataset, 20 labeled data samples were used to train these fully supervised models.

### 4.2 Results

Table 1 shows the performance of the proposed method in the COVID‐19 CT dataset. The results demonstrate that the proposed method outperforms in the segmentation task. In terms of the Dice metric, the proposed method improves by 0.76 compared to the well‐performing TUMT.^46^ In terms of Intersection over Union (IoU), the proposed method also achieves the best results, demonstrating higher accuracy in image segmentation. However, the segmentation performance of the model is slightly inferior in terms of MAE and FNR metrics, indicating that some target regions are misclassified as the background. Taking everything into account, the proposed method shows significant improvement in differentiating foreground and background compared to other semi‐supervised methods, demonstrating the powerful ability of using Transformer as the backbone for feature extraction and the effectiveness of applying the level set function in semi‐supervised methods.

**TABLE 1:** Comparison experimental results on the COVID‐19 CT dataset with the latest semi‐supervised networks.

Method	DSC(%)↑	IoU(%)↑	MAE(%)↓	FNR(%)↓
DCAN	71.19	56.98	3.32	24.36
PFNet	73.53	60.04	**2.81**	25.91
GGF^44^	73.60	60.28	3.14	23.14
TUMT	73.80	60.11	3.03	**20.68**
CLCNet^45^	71.74	57.93	3.36	23.28
ours	**74.56**	**61.26**	3.00	21.50

From Table 2, it can be observed that the proposed method also exhibits superior performance in the retinal vessel segmentation task. On this dataset, the proposed method surpasses other semi‐supervised methods in all metrics, indicating clear advantages when segmenting complex and morphologically diverse data such as blood vessels.

**TABLE 2:** Comparison experimental results on the DRIVE dataset with the latest semi‐supervised networks.

method	DSC(%)↑	IoU(%)↑	MAE(%)↓	FNR(%)↓
DCAN	79.31	65.79	5.06	25.42
PFNet	79.51	66.19	4.87	30.14
GGF	78.03	64.13	5.21	28.87
TUMT	77.89	64.01	4.96	32.58
CLCNet	77.93	64.12	4.94	32.63
ours	**79.79**	**66.7**	**4.77**	**24.78**

To further validate the effectiveness of the proposed method, a comparison is made with several state‐of‐the‐art Transformer‐based network structures, namely, Swin Transformer,^12^ Swin‐Unet,^24^ MedT,^30^ Pyramid Vision Transformer (PVT),^26^ MobileFormer,^47^ Convolutional Neural Networks Meet Vision Transformers (CMT),^48^ and NNUnet.^51^ These networks were trained using fully supervised methods, and the experimental results on the two datasets are presented in Table 3 and Table 4, respectively. Above experiments demonstrate that the proposed method achieves superior segmentation accuracy on different datasets, even when compared to fully supervised Transformer algorithms. Particularly, this method exceeds other methods in terms of the Dice and IoU measurements, indicating its ability to better capture image contextual information and semantic features, thereby improving the accuracy of medical image segmentation tasks. By extracting more comprehensive semantic and contextual information, this method can better handle the details and complexities in medical image segmentation tasks, resulting in better performance. Generally speaking, fully supervised methods often achieve better results due to the availability of ample data. However, by analyzing the data in Tables 1, 2, 3, and 4, we found that some semi‐supervised methods can achieve the same or even better results compared to fully supervised methods. We believe that aside from the amount of data, the way data is preprocessed and the design of the model's feature extraction structure also significantly impact the model's performance. Taking the COVID‐19 CT dataset as an example, it can be observed that semi‐supervised methods like Dynamic Convolution Adversarial Network (DCAN), Pulmonary Fibrosis Net (PFNet), Gated Feature Fusion (GFF), Tripled‐Uncertainty Guided Mean Teacher Model (TUMT), and Cross‐Level Contrastive Net (CLCNet) achieve segmentation performance comparable to fully supervised methods such as SwinTransformer, Swin‐Unet, and CMT, due to the carefully designed data augmentation methods or ingenious feature extraction module design. However, it can also be observed that two fully supervised methods, PVT and MobileFormer, did not achieve the expected segmentation results. This might be due to their lightweight designed structures. It is well known that how to balance efficiency and performance has always been a focal point in the field of deep learning. Currently, a lightweight and convenient model inevitably brings some performance degradation.

**TABLE 3:** Comparison experimental results on the COVID‐19 CT dataset with the latest Transformer‐based fully supervised network.

method	DSC(%)↑	IoU(%)↑	MAE(%)↓	FNR(%)↓
SwinTransformer	73.42	59.66	**2.88**	25.73
Swin‐Unet	71.29	57.22	3.04	28.41
PVT	70.03	55.59	3.13	31.00
MobileFormer	67.63	52.93	3.34	35.74
CMT	74.23	60.37	2.98	24.79
ours	**74.56**	**61.26**	3.00	**21.50**

**TABLE 4:** Comparison experimental results on the DRIVE dataset with the latest Transformer‐based fully supervised network.

method	DSC(%)↑	IoU(%)↑	MAE(%)↓	FNR(%)↓
SwinTransformer	76.89	62.54	5.53	26.24
Swin‐Unet	73.25	57.89	6.13	35.55
PVT	76.05	61.42	5.79	27.67
MobileFormer	78.87	65.20	4.95	26.13
CMT	76.81	65.20	5.72	26.89
ours	**79.79**	**66.7**	**4.77**	**24.78**

**FIGURE 3:**
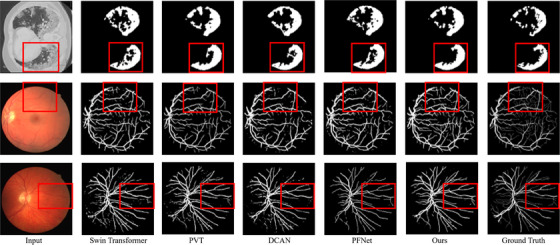
Partial visual results of our method, as well as several other fully supervised and semi‐supervised learning models, on the COVID‐19 CT test set and DRIVE test set.

Figure 3 displays visual results from part of the experiments. It can be observed that the proposed method achieves better segmentation compared to both semi‐supervised methods like DCAN^49^ and PFNer,^50^ and fully supervised methods like Swin Transformer and PVT. In the COVID‐19 dataset, the proposed method closely aligns with the labels, particularly in the highlighted regions shown in the boxes. In the DRIVE dataset, due to the complexity and numerous small branches in the segmented vessels, the segmentation results of other models often contain a significant amount of noise, while our method present fewer noise artifacts compared to other methods, along with an improvement in segmentation accuracy.

### 4.3 Ablation experiments

To validate the effectiveness of the proposed method and its structure, ablation experiments were conducted on the COVID‐19 CT dataset, specifically focusing on the loss function and the parallel Swin modules at each encoder. Table 5 displays the results of changing the loss function to confirm the impact of the signed distance map generated by the level set function in the regression

**TABLE 5:** Results of ablation experiments performed on the network structure on the COVID‐19 CT dataset.

method	DSC(%)↑	IoU(%)↑	MAE(%)↓	FNR(%)↓
L1	70.55	56.31	3.43	34.04
L2	72.05	58.03	3.25	29.72
ours	**74.56**	**61.26**	**3.00**	**21.50**

layer. Specifically, the loss functions were set as $L1_{\mathrm{ablation}}$ and $L2_{\mathrm{ablation}}$, formulas of two of them are as follows:

(12) $$L1_{\mathrm{ablation}}=\sum_{x_{i},y_{i}\in D}\left|\right|f_{1}\left(x_{i}\right)-C\left(y_{i}\right)|{|}^{2}-\frac{1}{p}\sum_{i=1}^{p}\log f_{i}\left(y_{i}^{*}\right),$$

(13) $$L2_{\mathrm{ablation}}=-\frac{1}{p}\sum_{i=1}^{p}\log f_{i}\left(y_{i}*\right).$$

$L1_{\mathrm{ablation}}$ removes the loss between signed distance maps and segmentation probability maps, while $L2_{\mathrm{ablation}}$ retains only the loss between labels and segmentation probability maps. From the experimental results, it can be observed that the segmentation results significantly deteriorated when the help of the signed distance map was lost. This demonstrates the role of the level set function and verifies the positive effect of using the signed distance map for semi‐supervised tasks.

Similarly, ablation experiments were performed on the backbone network, retaining Swin module branches with large window and Swin module branches with small window value. By comparing the results in Table 6 with the original method, it was found that combining the two Swin module branches with different window sizes in parallel indeed improved the performance of the network to some extent. This improvement can be attributed to the fact that the different window sizes combine global features and fine‐grained features, enriching the feature extraction process and enhancing the accuracy of segmentation.

**TABLE 6:** Results of ablation experiments performed on the network structure on the COVID‐19 CT dataset.

method	DSC(%)↑	IoU(%)↑	MAE(%)↓	FNR(%)↓
Big window Breach Only	71.19	57.25	3.41	23.47
Small window Breach Only	70.38	56.11	3.64	24.79
ours	**74.56**	**61.26**	**3.00**	**21.50**

## 5 CONCLUSIONS

In this paper, we have proposed a semi‐supervised medical image segmentation method based on the Transformer network. The method utilizes Swin modules with different window sizes to extract richer image features, while gradually reducing the image features during the encoding process, enabling the Transformer network to capture multi‐scale feature information. Additionally, a signed distance map generated by the level set function is incorporated to assist in improving the segmentation results. Specific loss functions are designed for labeled and unlabeled data based on the relationship between the regression layer and the segmentation output layer. The proposed method is evaluated on the COVID‐19 CT dataset and the DRIVE dataset. The results demonstrate that even when compared to fully supervised Transformer networks, the proposed method exhibits certain advantages, particularly in terms of segmentation accuracy, indicating its advancements. Although the parameter count in our current model architecture is relatively high, these sacrifices are worthwhile because they can achieve satisfactory segmentation results under reasonable conditions. In future work, we will strive to improve our approach to make it more efficient.

## AUTHOR CONTRIBUTIONS

**Xiaojie Huang**: Methodology; validation; funding acquisition; writing—original draft preparation. **Yating Zhu**: Software; validation; writing—original draft preparation. **Minghan Shao**: Software; writing—original draft preparation. **Ming Xia**: Software; formal analysis; resources; funding acquisition. **Xiaoting Shen**: Formal analysis; resources **Pingli Wang**: Conceptualization; supervision. **Xiaoyan Wang**: Conceptualization; methodology; funding acquisition; writing—review and editing. All authors have read and agreed to the published version of the manuscript.

## CONFLICT OF INTEREST STATEMENT

The authors declare no conflicts of interest.

## Data Availability

The data that support the findings of this study are openly available in the COVlD‐19 CT dataset at https://medicalsegmentation.com/covid19/, reference [51], and in the DRIVE dataset at https://drive.grand‐challenge.org/, reference [52].
